# Using multi-focus group method as an effective tool for eliciting business system requirements: Verified by a case study

**DOI:** 10.1371/journal.pone.0281603

**Published:** 2023-03-10

**Authors:** Robert M. X. Wu, Yongwen Wang, Niusha Shafiabady, Huan Zhang, Wanjun Yan, Jinwen Gou, Yong Shi, Bao Liu, Ergun Gide, Changlong Kang, Zhongwu Zhang, Bo Shen, Xiaoquan Li, Jianfeng Fan, Xiangqian He, Jeffrey Soar, Haijun Zhao, Lei Sun, Wenying Huo, Ya Wang

**Affiliations:** 1 School of Engineering and Technology, Central Queensland University, Sydney, Australia; 2 School of Geography, Shanxi Normal University, Taiyuan, China; 3 Shanxi Fenxi Mining Industry (Group) Co. Ltd, China; 4 Faculty of Science and Technology (Sydney Campus), Charles Darwin University, Darwin, Australia; 5 Shanxi Fenxi Mining Zhongxing Coal Industry Co. Ltd, China; 6 GENEW Technologies Co. Ltd, China; 7 Guangxi University, China; 8 School of Business, University of Southern Queensland, Australia; 9 Shanxi Kailain Technology Co. Ltd, China; University of Lisbon: Universidade de Lisboa, PORTUGAL

## Abstract

This research aims to explore the multi-focus group method as an effective tool for systematically eliciting business requirements for business information system (BIS) projects. During the COVID-19 crisis, many businesses plan to transform their businesses into digital businesses. Business managers face a critical challenge: they do not know much about detailed system requirements and what they want for digital transformation requirements. Among many approaches used for understanding business requirements, the focus group method has been used to help elicit BIS needs over the past 30 years. However, most focus group studies about research practices mainly focus on a particular disciplinary field, such as social, biomedical, and health research. Limited research reported using the multi-focus group method to elicit business system requirements. There is a need to fill this research gap. A case study is conducted to verify that the multi-focus group method might effectively explore detailed system requirements to cover the Case Study business’s needs from transforming the existing systems into a visual warning system. The research outcomes verify that the multi-focus group method might effectively explore the detailed system requirements to cover the business’s needs. This research identifies that the multi-focus group method is especially suitable for investigating less well-studied, no previous evidence, or unstudied research topics. As a result, an innovative visual warning system was successfully deployed based on the multi-focus studies for user acceptance testing in the Case Study mine in Feb 2022. The main contribution is that this research verifies the multi-focus group method might be an effective tool for systematically eliciting business requirements. Another contribution is to develop a flowchart for adding to Systems Analysis & Design course in information system education, which may guide BIS students step by step on using the multi-focus group method to explore business system requirements in practice.

## Introduction

During the COVID-19 crisis, more businesses planned to transform their businesses into digital businesses. Business managers face a critical challenge: they do not have adequate detailed system requirements and what is for digital transformation. Among many systems analysis approaches for understanding system requirements, focus group methods have been used to help elicit business information systems (BIS) for the past 30 years [1].

The literature identifies milestones in focus group studies: exploring attitudes in the 1960s, adopting qualitative marketing research since the 1970s, and eliciting BIS requirements from the 1990s. Goldman [2], for example, discussed the group interview technique to explore marketing, social science, and health research attitudes. Calder [3] and Morgan & Spanish [4] reported on focus groups as a natural tool for qualitative research. With origins in sociology, focus group studies were widely used in market research from the 1980s [1, 5]. Focus group studies were then applied to more diverse research applications from the 1990s [5]. However, up-to-date literature concerning the multi-focus group method about good research practices mainly focuses on a particular disciplinary field [6], such as social research [7, 8] and biomedical science [9]. The literature indicates that health research has often successfully used multi-focus group methods in under-studied research topics, such as assessing comprehensive geriatrics in health research [10, 11]. Limited research was identified that reported on using the multi-focus group method to elicit business system requirements: there is a need to fill this research gap.

This research aims to explore the multi-focus group method as an effective tool for eliciting business requirements systematically for BIS projects. A case study is conducted to verify that the multi-focus group method might effectively explore detailed system requirements to cover the Case Study business’s needs from transforming the existing systems into a visual warning system. The following sections describe the background, research method, systems analysis, systems design, conclusion, implications, contributions, limitations, and further research.

## Background

This research defines a multi-focus group method as more than two group interview sessions delivered for a project with the same facilitator and a group of participants.

### Features of the multi-focus group method

Peer-led focus groups are recognised as a means to help transform talk into action [12]. Focus group studies are dynamic, reflecting the participants’ comfortable talking [13]. The main significant feature of the focus group method over other research methods is that it can help achieve new or different insights. A Focus group study can have advantages in comparing many opinions [6, 14]. It allows for analyzing shared understandings and collective representation within different social clusters [15], contrasting research findings with existing literature, and even revealing different perspectives [6]. Focus groups may help participants identify and clarify their views synergistically [16], and gain new insights into a complex topic [6, 17].

The multi-focus group method enhances the three features compared to the single-focus group method. The first enhanced significant feature is to encourage more interactions. Generally, focus groups are conducted using a semi-structured discussion guide developed through an iterative process, whereby data from transcripts and researchers’ notes from the first focus group are examined and used to guide the discussion in subsequent focus groups [18]. The focus group method encourages interactive discussions among the participants [11]. The interaction among the participants is believed to help explore and clarify their experiences and views concerning the study’s aims [19]. The multi-focus group offers more opportunities for the participants to present discrepancies. Any opinions during the focus group sessions were discussed in the subsequent session until a consensus was reached, and the data were systematically rearranged into subcategories and categories [20].

The second feature is the opportunity multi-focus groups offer to generate abundant data: adequate peer-led focus group studies can generate rich research data [12, 21]. Multi-focus group studies can bring more and various experts’ perspectives together with common interests [11, 22–25], which may provide rich data from fruitful discussions [21] and help researchers to gather material as rich as possible [11].

The third enhanced feature of the method is that it can lead to a more comprehensive understanding. Each focus group session might conduct an unstructured discussion with a group of people and involve individuals with common expertise and interest [11, 24] enabling in-depth discussion among participants [26]. These studies open up the possibility of bringing various experts’ perspectives together and discussing central arguments with a breadth of accounts from participants [6, 17, 22, 23, 25]. They might gain a more comprehensive understanding by revealing the opinions and experiences of participants [22].

Based on the above three significant enhanced features- encouraging interaction, gathering abundant data, and leading comprehensive understanding, the multi-focus group method is especially suitable for investigating issues with no previous evidence, unstudied research topics, or less well-studied phenomena [20].

### Background of case study mine

**Fig 1:**
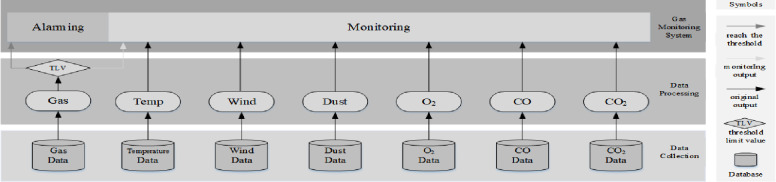
Current gas monitoring system in the case study mine. (Wu et al. 2021, p.3180) [46].

Shanxi Fenxi Mining ZhongXing Coal Industry Co. Ltd (ZhongXing) was selected as a Case Study mine. The existing gas monitoring system in the Case Study comprises two sub-systems: the alarming sub-system and the monitoring sub-system. The current alarming sub-system focuses on detecting real-time data obtained from methane gas sensors (called gas data in this paper). The gas monitoring system will alert the safety-responsive team if the gas data outputs reach the threshold limit value (TLV). The current monitoring sub-system monitors data obtained from gas sensors, temperature sensors, wind sensors, dust sensors, O_2_ sensors, CO sensors, and CO_2_ sensors. Data outputs of temperature, wind, dust, O_2_, CO, and CO_2_ are communicated to the monitoring system. Fig 1 shows the system framework of the current gas monitoring system in the Case Study mine.

The top management in Case Study mine had made a business plan with new requirements to upgrade the current gas monitoring system. A warning sub-system needed to be added to the existing gas monitoring system to improve the sensitivity and reduce the incidence of gas explosions and other adverse events. Business managers in Case Study mine faced a critical challenge: they did not have detailed system requirements or the requirements for transforming to a digital platform. This research uses the muti-focus group method to help elicit their requirements from transforming existing systems into digital visual warning systems.

### Research method

The multi-focus group method with unstructured discussion is used as a qualitative research method for eliciting system requirements to investigate less previous successful evidence and more understanding of system requirements for the Case Study mine project.

### Participants and settings

The optimal number of participants in focus group studies is between six and twenty [5, 27, 28]. The project leader takes responsibility for selecting participants from project stakeholders. These participants should be selected from different stakeholder groups, such as industry top management, business management, system development, and end-users.

### Data collection

System requirements for business applications were collected during multi-focus group sessions. The project leader should select two facilitators experienced in the focus group study method and knowledgeable about the conducted project background. The project leader attended all focus group sessions. Two facilitators collaborated and conducted all sessions. The facilitators used a loosely constructed set of relevant questions for facilitating each focus group session [27]. At the beginning of the first focus group session, the facilitators encouraged group interaction by inviting the participants to describe their experiences and understandings of the project [24]. The facilitators asked participants to introduce their field experiences to those from industry companies and research interests to those from academic institutions. The facilitators then explained why a focus group method was chosen and encouraged the participants to address each other [19]. During the focus group session, facilitators posed follow-up questions to encourage crosstalk among participants [19, 29].

Brainstorming approaches might be used during each focus group meeting. The brainstorming discussions might guide the researchers in identifying and prioritizing the system requirements [30]. The facilitator(s) used prompts to elicit specific hypotheses from the literature when participants did not mention them [31]. After the focus group session, the facilitator(s) debriefed with other research team members to identify and note the conversation’s initial impressions and critical points [32].

The facilitator(s) will examine data, questions, and researchers’ or discussion notes from each focus group. The outcomes will guide the discussion in subsequent focus group sessions [18]. Similarly, the procedures will be repeated in the following sessions. Each session lasted an average of between one to two hours.

### Multi-focus group discussions

The open-question discussions were delivered to all focus groups. All feedback and conversations were recorded and transcribed verbatim. This research did not use audio or video-record data to encourage participants to share their experiences in a more stress-free and relaxing environment. Data were analyzed using an inductive content approach to analyze focus group transcripts. This approach may identify patterns from focus group discussions [6, 14].

### Participant consent

The participants were informed thoroughly in written form and verbally about the aim of the study [11]. All participants were told that personal information and discussions would be kept confidential [33] and how confidentiality would be handled [11]. They were also told that their personal information would not be publicly available due to privacy and ethical restrictions [34]. Only the research team members could access the original interview files and transcripts [35]. They were also advised that focus group data would be analyzed and published in a research journal [33, 35, 37].

### Ethical considerations

This research was approved by the Shanxi research committee office (ID: 201809fx03) under ethical research considerations managed by a state government. Ethical approval is not always required for the focus group interview [11]. The invitation to participate emphasises that participation is voluntary [11] and they could withdraw from the focus group sessions at all stages of the research without giving a reason without repercussions [19, 24].

## Systems analysis

Systems analysis systematically focuses on capturing the logic and identifying the requirements of the business environment [36]. This section reports on a mining application to demonstrate how to use the multi-focus group method as a development tool for conducting systems analysis to identify the system requirements in the Case Study mine.

### Participants and settings

**Fig 2:**
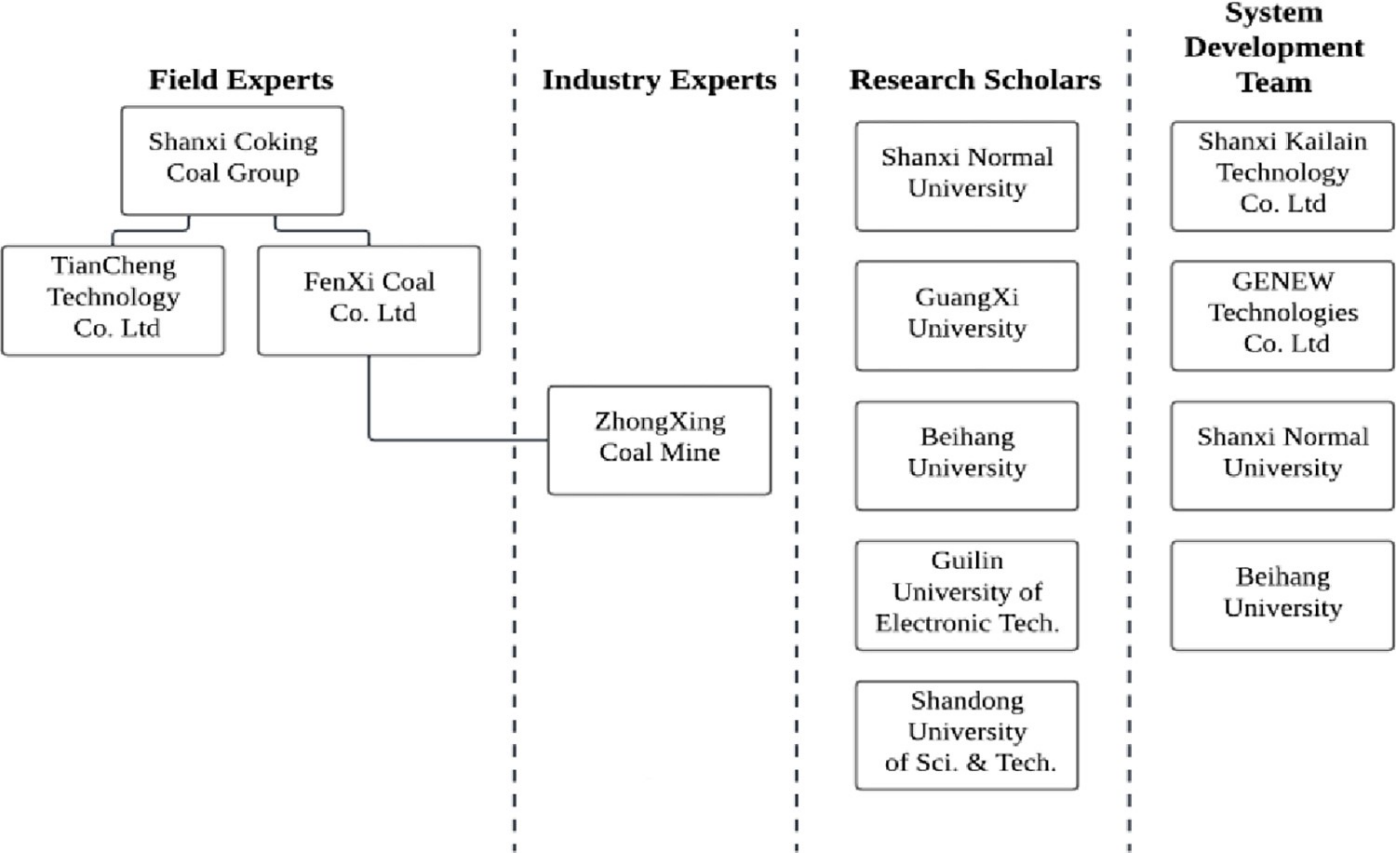
Organizational chart of the project stakeholder.

Fig 2 shows the organizational chart of the project stakeholders in the Case Study. They include four clusters: field experts, industry experts, research scholars, and the system development team. All experts were thoroughly informed in written form and verbally.

During the first meeting they attended, the aim and background of this project with the approved project number were introduced. All participants were asked to keep all data confidential, including personal information and discussion documents. The research team members only accessed them. The project sponsor and founder recommended the field experts and industry experts at the executive and senior levels. They were from Shanxi Coal Group Co. Ltd, TianCheng Technology Co. Ltd (TianCheng), and FenXi Coal Co. Ltd (FenXi). Industry experts were from the Case Study mine–Shanxi Fenxi Mining ZhongXing Coal Industry Co. Ltd (ZhongXing). Shanxi Coking Coal Group Co. Ltd. was ranked 485th in the 2020 Fortune Global 500 and was China’s largest coal mining company and coking coal supplier [37]. TianCheng and Fenxi were owned wholly by Shanxi Coking Coal Group. ZhongXing was owned entirely by Fenxi.

Research scholars were from five of China’s universities, including the School of Geographical Sciences at Shanxi Normal University, the School of Resources, Environment and Materials at Guangxi University, the School of Software at Beihang University, the Center for Cloud Computing at the Guilin University of Electronic Technology, College of Energy and Mining Engineering at the Shandong University of Science and Technology, and College of Computer Science and Engineering at the Shandong University of Science and Technology.

The system development teams included system developers from Shanxi Kailain Technology Co. Ltd, GENEW Technologies, the School of Geographical Sciences at Shanxi Normal University, and the School of Software at Beihang University.

### Data collection

Data collection and analysis started in January 2018 and ended in November 2020 with a span of three years. Data collection and analysis involve five processes and eleven focus group sessions. They include the first process—identifying the requirements (including two focus group sessions), the second process–planning the project (focus group session 3), the third process–discovering the project needs (focus group sessions 4 to 7), the fourth process–developing the system (focus group sessions 8 and 10), and the fifth session–testing and integrating the system components (focus group session 11).

The project team leader facilitated all focus group studies and appointed two senior members as facilitators, including one field expert and one research scholar. Brainstorming was conducted for each focus group session to guide the participants in identifying and prioritizing the project requirements during the first focus group session. The project team leader and two co-facilitators examined collected data and discussion notes from the first focus group and then forwarded the discussion outcomes to the following session. Another brainstorming then focused on them during the next session. The results of the previous session were also examined and sent to the next session for further brainstorming discussions.

The participants included field experts (34.09%, 60 out of 176), industry experts (32.95%, 58 out of 176), research scholars (18.18%, 32 out of 176), and system developers (14.77%, 26 out of 176) (Table 1). The focus group sessions 1 to 10 utilized face-to-face workshops at four places at ZhongXing, FenXi, TianChen, and Shanxi Coal. The last focus group session was delivered through online discussions during the COVID-19 lockdown period. The number of participants involved in each focus group session research was between 7 and 21.

**Table 1:** Descriptive information on focus group studies.

Project Processes	Focus Group Sessions			Field Expert	Industry Expert	Research Scholar	System Development Team	Sum
	No	Date	Location					
**Identifying the requirements**	1	23 Jan,18	ZhongXing	8	7	3	3	21
	2	26 Jan,18	ZhongXing	6	7	3	3	19
**Planning the project**	3	27 Jan,18	TianCheng	13		3	3	19
**Discovering the Project’s needs**	4	19 Jun,18	Shanxi Coal Group	15	3	2		20
	5	26 Jun,18	TianCheng	7	3	4		14
	6	04 Dec,18	ZhongXing	1	10	5	2	18
	7	05 Dec,18	FengXi	4	3	5	2	14
**Developing the system**	8	08 May,19	ZhongXing	2	6	2	4	14
	9	22 Aug,19	ZhongXing	2	11	2	6	21
	10	22 Nov,19	FengXi	2	4	1	2	9
**Testing and integrating components**	11	10 Nov,20	Online		4	2	1	7
**Total**				60	58	32	26	176

Focus group studies had a span of three years:. the focus group sessions had different focus groups in each phase of the studies. The main reason for this was that the project stakeholders became more understanding of the business’s needs and systems requirements at the end of the study. For example, session 1 had the most significant number of participants (21). Session 10 had the second smallest number of participants (9). Session 11 had the smallest number of participants (7). Another reason was that participants were invited based on the project’s needs. Sessions 4 and 5 did not request any development team members because all requirements had been well-discussed in the previous process phase–identifying the requirements and planning the project.

After interviewing groups 4 and 5, the project leader and facilitators realized that it was necessary to include System Development Team participants in the following sessions because all modified or added requirements involved system design. The third reason was that several experts, scholars, and development team members changed due to management, employment, and position changes. A few members did not attend consequence studies because of their health statuses due to the COVID-19 crisis.

73 participants in total were involved in this research. They included field experts (47.85%, 35 out of 73), industry experts (21.92%, 16 out of 73), research scholars (13.70%, 10 out of 73), and system developers (16.44%, 12 out of 73). Table 2 shows the characteristics of the interviewed participants (n = 73) in all focus group sessions, including their gender, age, and level of education.

**Table 2:** A description of demographic data collected.

	Variable	Field Expert (47.85%)	%	Industry Expert(21.92%)	%	Research Scholar(13.70%)	%	System Development Team(16.44%)	%	SUM	Total
Gender	Female	6	8.22%	0	0.00%	2	2.74%	2	2.74%	10	73
	Male	29	39.73%	16	21.92%	8	10.06%	10	13.70%	63	
Age	20–34	3	4.11%	0	0.00%	3	4.11%	3	4.11%	9	73
	35–49	30	41.10%	16	21.92%	7	9.59%	5	6.85%	58	
	>50	2	2.74%	0	0.00%	0	0.00%	4	5.48%	6	
Education	Junior college	0	0.00%	1	1.37%	0	0.00%	1	1.37%	2	73
	Undergraduate	0	0.00%	14	19.18%	0	0.00%	1	1.37%	15	
	Postgraduate	33	45.21%	1	1.37%	3	4.11%	7	9.59%	44	
	doctor	2	2.74%	0	0.00%	7	9.59%	3	4.11%	12	

### Reliability and validity

Ensuring the rigor of data analysis, two facilitators independently reviewed and analyzed the feedback and transcripts to avoid inconsistent meaning or expression and reduce ambiguities, distortions, and bias. One was a senior field expert from ZhongXing–the end-user company of this project. Another facilitator was a senior researcher from the School of Resources, Environment, and Materials at Guanxi University, China.

All focus group sessions invited multiple participants (between 7 and 21) (Table 1) and four clusters (field experts, industry experts, research scholars, and the system development team) (Fig 2). This strategy was intended to reduce researchers’ bias and to ensure the categories were data-driven [10]. Another strategy was to discuss discrepancies in the subsequent session until a consensus was reached. The project manager attended all sessions. Two facilitators collaborated and conducted all sessions. Facilitators analyzed each other’s data independently and aimed to improve the study’s reliability [20]. During focus group sessions, facilitators should encourage contributions from all participants to be clear, which increases the reliability of the results [38]. They ensured that all discrepancies were covered until a consensus was reached.

In this research, the multi-focus group sessions were conducted to different project processes, such as identifying the requirements in two focus group sessions (sessions 1 and 2), discovering the project’s needs in four sessions (sessions 4–7), and developing systems in three sessions (sessions 8–10).

### Systems requirements collected

Twenty-eight questions (Q1 to Q28) were collected and discussed from focus group sessions 1 to 11. They covered business requirements to ensure that the proposed solutions might meet organizational goals. They were divided into four clusters (S1 Appendix), including Data Acquisition (DA), Data Isolation (DI), Alarming and Early Warning (AEW), and System Interface Design (SID).

S2 Appendix shows the linkages between 28 issues, suggestions, and questions. They include Data Acquisition (DA1 to DA6), Data Isolation (DI1 to DI8), Alarming and Early Warning (AEW1 to AEW8), and System Interface Design (SID1 to SID6).

## Systems design

Systems design intends to provide IS solution designs that fit the business environment and address the identified needs [36]. Following the above procedure of systems analysis, systems design has proposed the solutions to the Case Study mine.

### Systems design-proposed solutions to focus group studies

The following seven solutions (S1 to S7) were proposed to fit the Case Study mine’s needs for the innovative system. They were designed to address and solve all 28 issues and suggestions (S2 Appendix) identified in the systems analysis during the focus group sessions.

#### Solution 1 (S1) was proposed to cover DI1

S1 built a data warehouse management to cover DI1. The data warehouse management system was proposed to be developed and interact with various databases from eight primary types of coal mine monitoring systems deployed in the Case Study mine, including a Gas Monitoring system, Pumping Monitoring System, Dynamic Roof Monitoring System, Hydrological Monitoring System, Gas Extraction System, Power Monitoring System, Water Sump Monitoring System, and Tube Bundle Monitoring System.

#### Solution 2 (S2) was provided to solve DA1 and DA2

S2 provided the module of data pre-processing for eliminating anomaly data and extreme values to solve DA1 (anomaly data) and DA2 (extreme data). Data pre-processing consists of transforming the data values of a specific dataset, aiming to optimize the information acquisition and process while there is a significant contrast between the maximum and minimum values of the dataset, so normalizing the data minimizes the complexity of the algorithm for its corresponding processing [39]. Data processing was proposed to cover three data cleaning procedures: eliminating extreme values, eliminating outliers, and standardizing data.

#### Solution 3 (S3) was conducted to ensure covering DA4 and DA5

S3 conducted data analysis on the reliability and validity to examine any sensor changes. It ensured covering DA4 (sensors’ physical address changed at the same working-face) and DA5 (sensors’ physical address changed to a different working-face).

#### Solution 4 (S4) undertook correlation analysis to probe DA3, DA6, DI2, DI3, DI4, DI5, DI7, DI8, AEW1, AEW2, AEW3, AEW4, AEW5, AEW6, AEW7, AEW8, and SID2

S4 undertook correlation analysis to probe whether a strong relationship or evidence existed regarding DA3, DA6, DI2, DI3, DI4, DI5, DI7, DI8, AEW1, AEW2, AEW3, AEW4, AEW5, AEW6, AEW7, AEW8, and SID2. This solution analyzed correlation to uncover hidden patterns, incorporate correlations between coal monitoring systems, and confirm a strong relationship between gas data and other sensors’ outputs. As a quantitative research method, correlational research results can inform causal inferences and evidence-based practice and subject them to an experimental study [40]. This method can be used in any analysis that does not wish to manipulate the investigated independent variables [41].

When the correlational research method is adopted, correlation analysis confirms a strong relationship between the sensor data. It can give a solid indicator to interpret a robust nonlinear relationship between nonlinear-dependent variables [42]. Therefore, correlational research studies can provide invaluable information about what future research may be required to investigate the variables shown to be correlated with the outcomes or attributes previously studied [41]. The consequences of integrating correlation analysis of data might improve the sensitivity of current gas warning systems and reduce the incidence of explosions and other adverse events.

The correlation coefficient was used to evaluate and measure the correlation between two pairs of input and output variables. The recent research found that there was no standard classification of the correlation coefficient scales and suggested six scales classifying the degree and magnitude of correlation as great (between ± 0.9 and ± 1), very good (between ± 0.75 and ± 0.89), good (between ± 0.5 and ± 0.74), fair (between ± 0.3 and ± 0.49), poor (between ±0.0 and ± 0.29), and no correlation (zero) [43]. This research follows a correlation value of ±0.3 or above to indicate the existence of a correlation between two variables.

#### Solution 5 (S5) was proposed to answer the question of DI6\

S5 followed activated decision rules to answer the question of DI6 (Integrating an early warning system into the gas monitoring system). It proposed integrating the warning sub-system into the current gas monitoring system with an early warning decision-making rule [43]. The warning system might use the weighted indexing measurement in risk assessments [44].

#### Solution 6 (S6) was explored to solve SID1 and present SID3

S6 explored an interface view sub-system added into the warning sub-system to solve SID1 (Lack of data visualization to the system interface) and presented SID3 (Adding the various sensors’ location into the system interface). As an emerging and complementary data analysis tool, data visualization may envision the relationships and then communicate those relationships convincingly to others [45].

25 visualization approaches have been discussed in Q1 publications until 2021 [46]. They include Bar Chart, Bubble Map, Dimension Hierarchies, Geo-Spatial maps, Glyphs-based techniques, Heatmaps, Histograms, Line Charts, Network Diagrams, Parallel Coordinates Plot, Pie Chart, Pixel-based techniques, Polar Coordinate Plots, Radial visualizations, Radar Chart, Sankey Chart, Scatter Plot, Stacked Graph, Sunburst, Table Lens, Topological Hierarchies, TreeMap, Typographic, Tag Clouds, and Weather Map. Among these approaches, eight data visualization tools are used to determine the outliers. They include Line Charts, Geo-Spatial maps, Glyphs-based techniques, Parallel Coordinates Plots, Pixel-based techniques, Redial visualizations, Scatter Plots, and TreeMap [47]. A Sankey Chart has the highest performance, usability, and superior satisfaction [48].

However, up-to-date research highlights that no single approach is well-performed for developing a visual warning system [43]. A new visualization approach needs to be explored to meet the Case Study mine’s requirement to design the data visualization interface for the system. The system should visually locate all sensors and alarm the safety-response team for anomaly data.

#### Solution 7(S7) was proposed to comply with SID4, SID5,and SID6

S7 proposed an Emergency Alert Mobile Warning System for sending mobile text messages to comply with SID4 (Sending warning texts via mobile devices), SID5 (Setting several warning levels for warning to the relevant staff), and SID6 (Recording all warning texts sent via mobile devices). The warming levels were set within a defined management team about warning emergencies. A voice message should also be sent to alert the safety-responsive team in case of an emergency. However, a mobile phone text alert should only focus on identified crisis warnings. Receiving too many alerts about “minor” incidents may degrade the system’s impact on behavior [49]. The records of the staff sending and receiving text messages should be backed up for further investigation.

### A three-layer architecture for presenting the solutions to the proposed system

**Fig 3:**
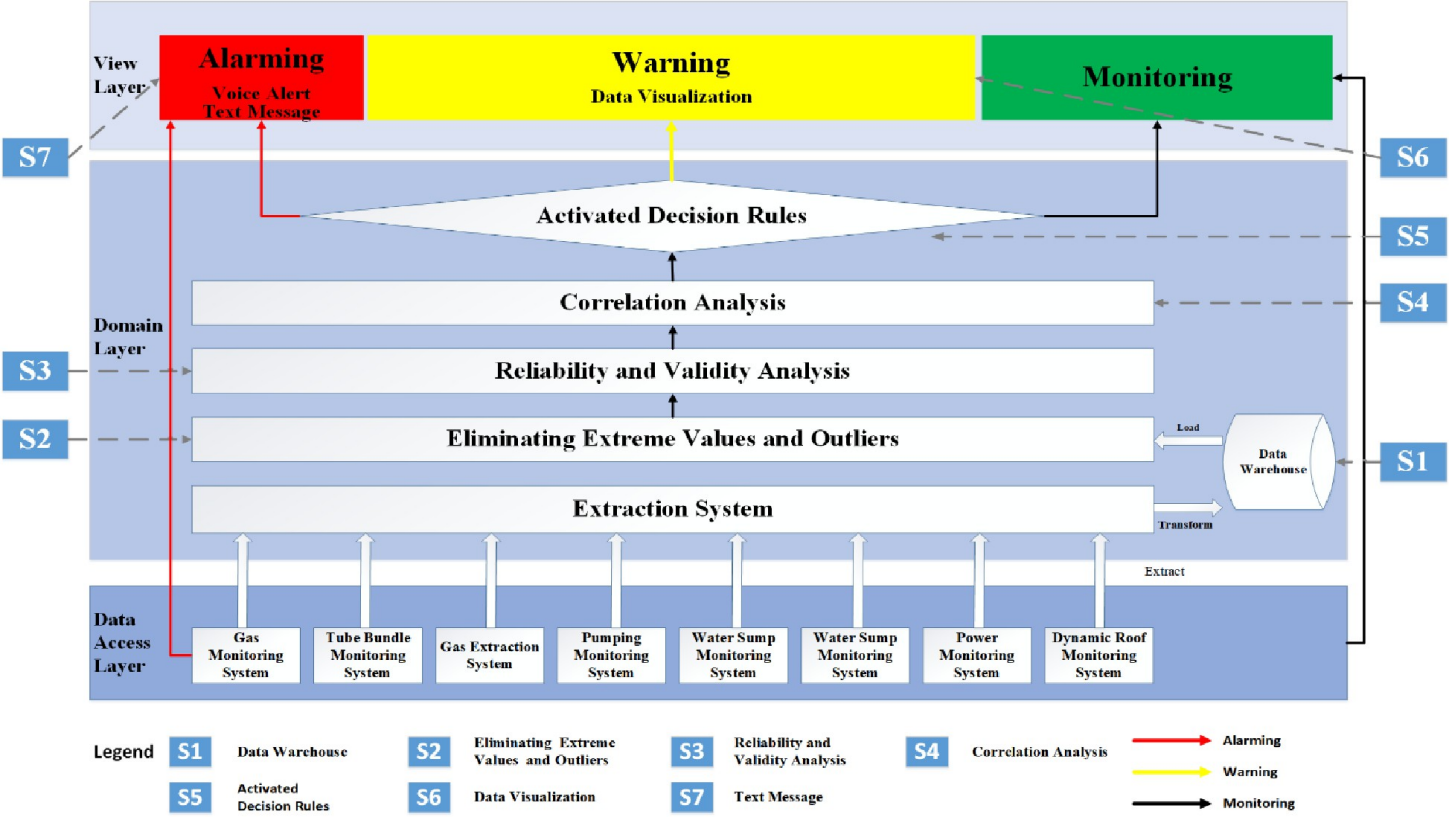
A three-layer architecture for demonstrating the proposed system.

Based on the above discussions, a three-layer architecture was developed to demonstrate the proposed seven solutions (Fig 3). It comprised three layers (data access, domain, and view layer) to illustrate the system’s architectural design. The data access layer incorporated eight coal mine monitoring systems. The domain layer included the extraction system, data warehouse, elimination of extreme values and outliers, reliability and validity analysis system, correlation analysis system, and activated decision rules. The view layer comprised three sub-systems: alarming, warning, and monitoring.

## Conclusion and implications

### Conclusion

This research aimed to explore the multi-focus group method as an effective tool for eliciting business requirements systematically for BIS projects. A case study was conducted to verify whether the multi-focus group method might effectively elicit system requirements as a tool to cover the business’s needs from transforming the existing systems into a visual warning system. The multi-focus group method was used to collect the issues and suggestions for the proposed system, including 11 focus group sessions with 176 participants in total, including field experts (60), industry experts (58), research scholars (32), and system developers (26). Data collection and analysis involve five processes and eleven focus group sessions. They include the first process—identifying the requirements (including two focus group sessions), the second process—planning the project (focus group session 3), the third process—discovering the project needs (focus group sessions 4 to 7), the fourth process—developing the system (focus group sessions 8 and 10), and the fifth session—testing and integrating the system components (focus group session 11).

To summarize the research, the multi-focus group method outstands three significant enhanced features compared to the focus group method, including encouraging interaction, gathering abundant data, and leading comprehensive understanding. The research outcomes verify that the multi-focus group method might effectively explore detailed system requirements to cover the business’s needs. This research identifies that the multi-focus group method is especially suitable for investigating less well-studied, no previous evidence, or unstudied research topics.

### Implications for IS education

There is a practical implication for IS higher education. The IS study offers students the bridge for two extensive bodies of knowledge: understanding information technology management and applying business processes and practices [50]. Due to rapid technological changes, business school curricula need to be updated with input from industry organizations [51] and develop opportunities for the students to have real-world, practical, relevant experience and make a case for the value of IS courses [50]. They impel business schools to undergo periodic incremental and occasional radical changes in IS curricula and reflect changing business practices [52].

Rapid technological changes also push higher education to well-prepare IS students with training in critical thinking, problem identification, and problem-solving skills [53, 54]. Systems requirement analysis is a core process for developing BIS applications [55, 56]. As a fundamental unit of IS education programs, Systems Analysis and Design (SA&D) involves understanding business requirements to ensure that the IS solutions are developed to meet organizational goals [56]. SA&D familiarises IS students with the methodologies, tools, and methods for developing IS applications [57].

A flowchart is developed that might be added to the SA&D course to guide BIS students step by step on using the multi-focus group method to explore business system requirements for an industry project effectively (S4 Appendix). They include being less knowledgeable of their needs, solving unstudied research or project, implying a semi-structured discussion to encourage interaction, leading to a comprehensive understanding, and generating rich data.

## Contributions, limitations, and further research

The main contributions can be stated as follows:

- This research verifies that the multi-focus group method might be an effective tool for systematically eliciting business requirements (S3 Appendix).
- A flowchart is developed that might be added to the SA&D course to guide BIS students step by step on using the multi-focus group method to effectively explore business system requirements for an industry project (S4 Appendix).

The main limitation was that the research outcomes mainly focused on systems analysis and design for the mining IS application in a Case Study mine company. Further research should be performed to examine whether a multi-focus group study method might be adopted in other industry sectors. Another limitation was that this research mainly focused on recent literature published after 2016 to present the latest five years. The reason was that a comprehensive review of the multi-focus group method was beyond the scope of this research. There is a need to conduct further research on a systemic review to understand better using a multi-focus study method to elicit system requirements in IS projects.

There is also a need to undertake further research that investigates demographic data and compares age, sex, employment, level of education, and position. The research outcomes would be valuable for forming participants and settings in further focus group studies.

## Supporting information

S1 Appendix Twenty-eight questions divided into four clusters. (DOCX)

S2 Appendix Issues and suggestions collected during focus group studies. (DOCX)

S3 Appendix Flowchart of The SDLC processes, focus group studies, systems analysis, and systems design. (DOCX)

S4 Appendix A flowchart as a guide to using multi-focus group method. (DOCX)
