# Supplementary material for: Using multi-focus group method as an effective tool for eliciting business system requirements: Verified by a case study
Source: PLoS One. 2023 Mar 10;18(3):e0281603. doi: 10.1371/journal.pone.0281603 (PMC10027421; doi:10.1371/journal.pone.0281603)
Supplement: S1 Appendix — (DOCX) [file pone.0281603.s001.docx]

**S1 Appendix: Twenty-eight questions divided into four clusters**

| - Cluster 1 - Data Acquisition (DA) |
| --- |
| Q1: There were issues obtaining data from the sensors, such as inaccuracy, extreme data, and anomaly data.  Q2: Current monitoring systems focused only on real-time data streams and did not utilize any historical data records. |
| Q3: If the sensors' physical address was changed at the same coal mining working-face, how did the data analysis impact it?  Q4: If the sensors' physical address was changed to a different coal mining working-face, how did the data analysis impact it? |
| Q5: How to utilize different sensors' data from various systems? |
| - Cluster 2 - Data Isolation (DI) |
| Q6: Each monitoring system was run independently and did not be connected to other systems.  Q7: The data between different monitoring systems were run separately. No correlation analysis was conducted.  Q8: The current monitoring system focused on the gas monitoring system. How did the impacts on various systems add to the gas monitoring system?  Q9: If the sensors' physical address was changed within the same coal mining working-face, how did the data analysis impact it?  Q10: If the sensors' physical address was changed to a different coal mining working-face, how did the data analysis impact it?  Q11: Integrating an early warning system into the gas monitoring system is necessary.  Q12: There is a need to conduct a correlation analysis between the data obtained from the wind and gas sensors.  Q13: There is a need to conduct a correlation analysis between the power and gas monitoring systems. |
| - Cluster 3 - Alarming and Early Warning (AEW) Requirements |
| Q14: We should set several alarming or warning levels, such as gas concentration fluctuation (exceeding 10%) and the fluctuation duration. |
| Q15: Current gas monitoring systems did not provide early warning notices and did not conduct correlation analysis. They did not explain the cause-and-effect reasons why gas data exceeded TLV. |
| Q16: The reports for reasons of anomaly data or exceeding TLV were based on personal experiences. The current gas monitoring system cannot predict early warning, so the safety-responsive team may immediately take relevant actions. If the gas data exceeded the TLV, the decisions were made based on personal experiences. The gas monitoring system was run independently and did not be connected to other systems. |
| Q17: Prediction of the early warning system should be based on various critical factors in the coal mining working-faces rather than just focusing on TLV. |
| Q18: The current data analysis system focused mainly on machine learning outputs and ignored human experts' analysis.  Q19: According to the current gas monitoring system, about 80% of the alarming cases were suddenly. Others were transmitted from quantity to quality changes.  Q20: There is a need to provide text messages for causal analysis.  Q21: How to predict the anomaly data? Most of the data obtained from the current gas monitoring system were normal and not anomaly data.  Q22: There is a need to explain the cause-and-effect reasons why gas data exceeded TLV and provide the suggested solutions. |
| - Cluster 4 - System Interface Display (SID) |
| Q23: The system interface of the current monitoring system was lack of data visualization.  Q24: Both wind and air volume should be added to the system interface.  Q25: Adding the location of the various sensors into the system interface.  Q26: There is a need to send early warning messages by text to mobile.  Q27: Several levels should be set for mobile alarms or warnings to other staff.  Q28: All text of alarm and warning to the relevant staff should be recorded. |
