# Supplementary material for: Using multi-focus group method as an effective tool for eliciting business system requirements: Verified by a case study
Source: PLoS One. 2023 Mar 10;18(3):e0281603. doi: 10.1371/journal.pone.0281603 (PMC10027421; doi:10.1371/journal.pone.0281603)
Supplement: S2 Appendix — (DOCX) [file pone.0281603.s002.docx]

**S2 Appendix: Issues and suggestions collected during focus group studies**

| **Question Cluster** | **Issues and Suggestions** | **Questions Collected** |
| --- | --- | --- |
| **Data Acquisition** | DA1: Anomaly data. | Q1 |
|  | DA2: Extreme data. | Q1 |
|  | DA3: Focusing on real-time data streams only and not utilizing historical records. | Q2 |
|  | DA4: Sensors' physical address was changed at the same working face. | Q3 |
|  | DA5: Sensors' physical address was changed to a different working face. | Q4 |
|  | DA6: Utilizing different sensors' data from various systems. | Q5 |
| **Data Isolation** | DI1: Systems isolation. | Q6, Q7 |
|  | DI2: Data isolation. No correlation analysis between data obtained from different systems. | Q7 |
|  | DI3: Impacts on various systems add to the gas monitoring system | Q8 |
|  | DI4: Impacts on the data analysis between different sensors at the same coal mining working-face. | Q9 |
|  | DI5: Impacts on data obtained from various types of sensors at different coal mining working-face. | Q10 |
|  | DI6: Integrating an early warning system into the gas monitoring system. | Q11 |
|  | DI7: Correlation analysis between the wind data and gas data. | Q12, Q16 |
|  | DI8: Correlation analysis between the power monitoring system and gas monitoring system. | Q13, Q16 |
| **Alarming and Early Warning Requirements** | AEW1: The setting of different warning levels, such as 10% increased, periods of fluctuation. | Q14 |
|  | AEW2: Lack of early warning. | Q15, Q16 |
|  | AEW3: Lack of correlation analysis. | Q15,Q17,Q21 |
|  | AEW4: Lack of explanation of the cause-and-effect reasons why gas data exceeded TLV. | Q15, Q16, Q20, Q22 |
|  | AEW5: Decision-making based on personal experiences. | Q16 |
|  | AWE6: Focused mainly on machine learning outputs and ignored the analysis of human experts. | Q18 |
|  | AWE7: About 80% of the alarming cases were suddenly. Others were transmitted from quantity to quality changes. | Q19 |
|  | AWE8: Provide the suggested solutions. | Q20,Q22 |
| **System Interface Design** | SID1: Lack of data visualization of the system interface. | Q23 |
|  | SID2: Both wind and air volume should be added to the system interface. | Q24 |
|  | SID3: Adding the location of the various sensors into the system interface. | Q25 |
|  | SID4: Sending warning texts via mobile devices. | Q26 |
|  | SID5: Setting several warning levels for warning to the relevant staff. | Q27 |
|  | SID6: Recording all warning texts send via mobile devices. | Q28 |
