# Supplementary material for: Using multi-focus group method as an effective tool for eliciting business system requirements: Verified by a case study
Source: PLoS One. 2023 Mar 10;18(3):e0281603. doi: 10.1371/journal.pone.0281603 (PMC10027421; doi:10.1371/journal.pone.0281603)

**S3 Appendix: Flowchart of the SDLC Processes, Focus Group Studies, Systems Analysis, and Systems Design**


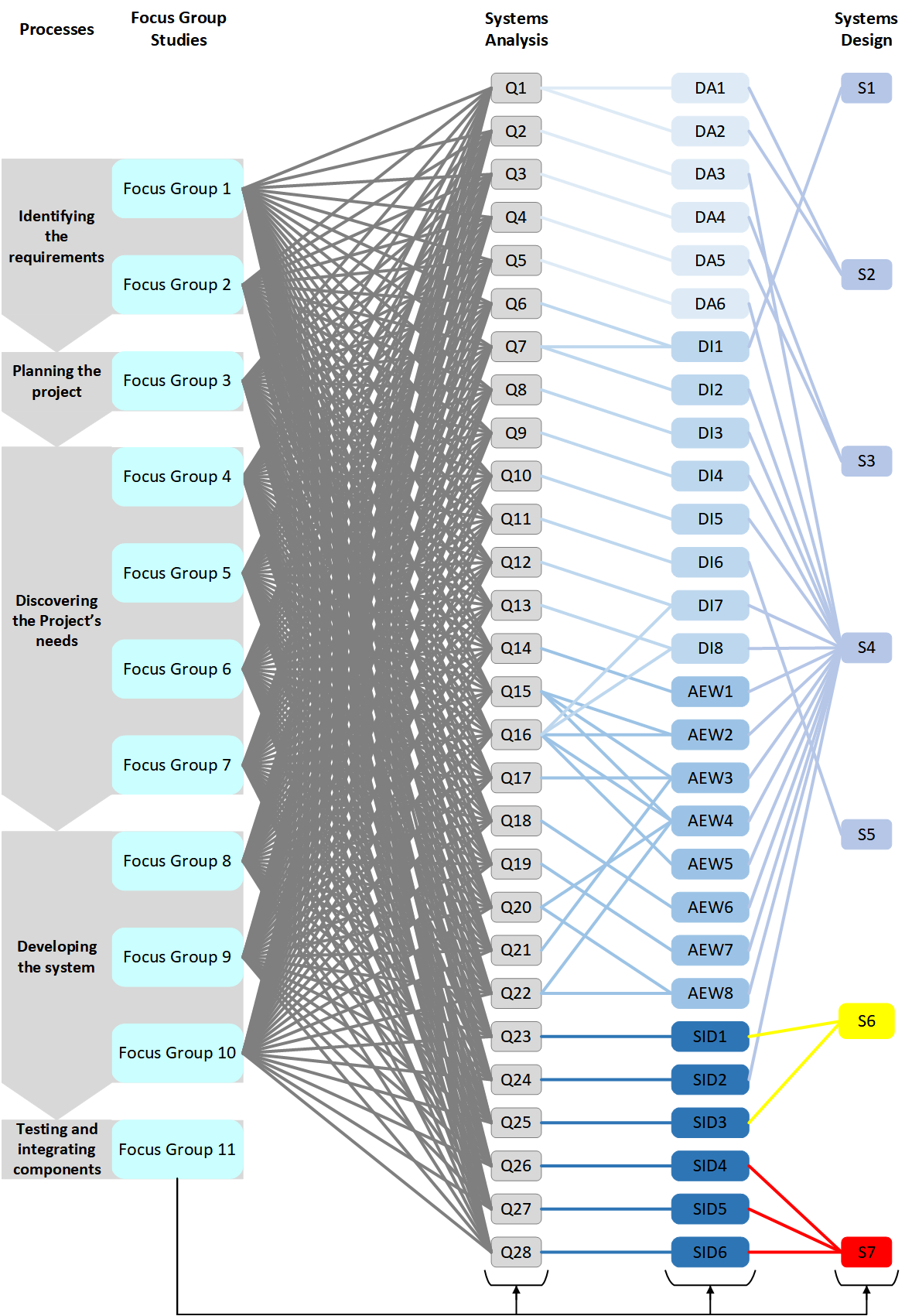

Supplement: S3 Appendix — (DOCX) [file pone.0281603.s003.docx]
