# Supplementary figures and images for: Using multi-focus group method as an effective tool for eliciting business system requirements: Verified by a case study
Source: PLoS One. 2023 Mar 10;18(3):e0281603. doi: 10.1371/journal.pone.0281603 (PMC10027421; doi:10.1371/journal.pone.0281603)

**S4 Appendix. A Flowchart as A Guide to Using Multi-Focus Group Method.**


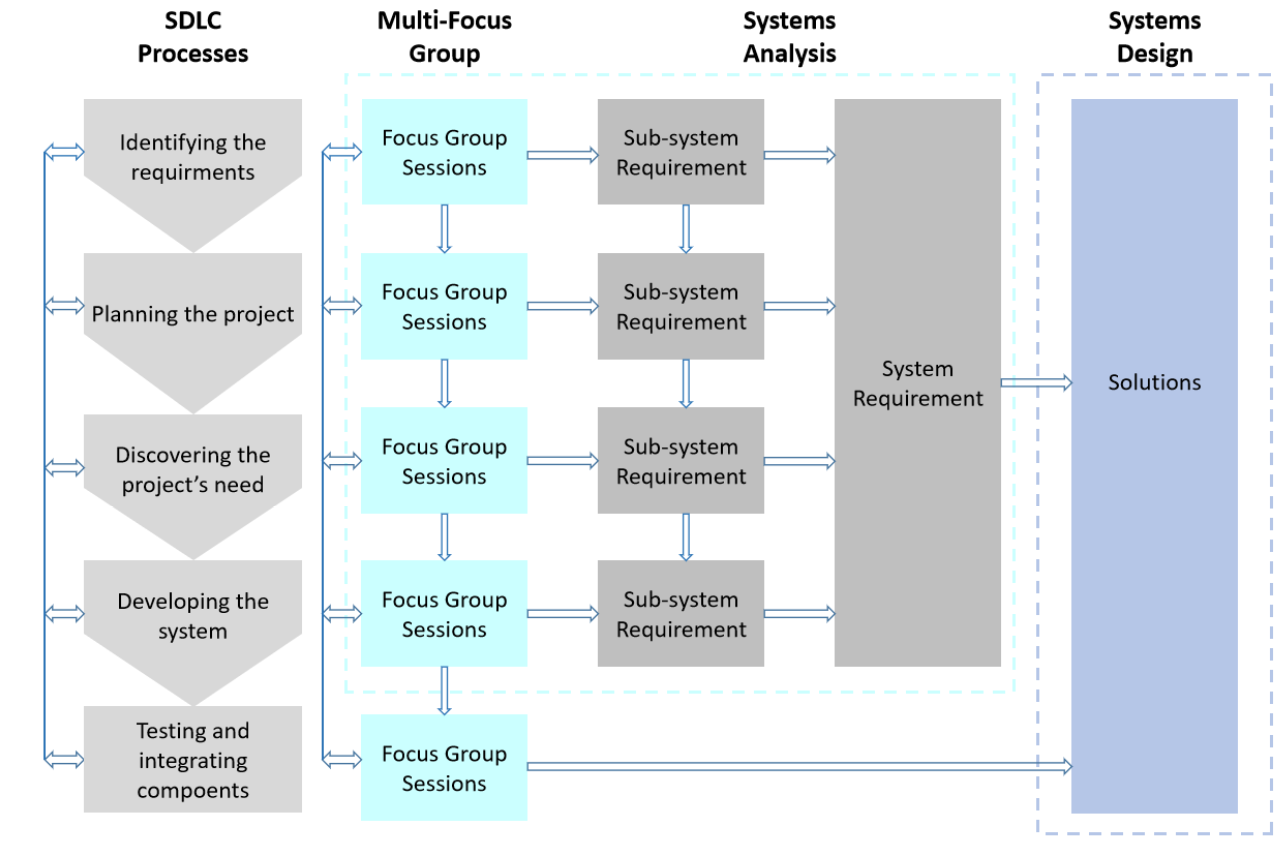

Supplement: S4 Appendix — (DOCX) [file pone.0281603.s004.docx]
